# The Unique Evolutionary Trajectory and Dynamic Conformations of DR and IR/DR-Coexisting Plastomes of the Early Vascular Plant Selaginellaceae (Lycophyte)

**DOI:** 10.1093/gbe/evz073

**Published:** 2019-04-01

**Authors:** Hong-Rui Zhang, Qiao-Ping Xiang, Xian-Chun Zhang

**Affiliations:** 1 State Key Laboratory of Systematic and Evolutionary Botany, Institute of Botany, The Chinese Academy of Sciences, Beijing, China; 2 University of Chinese Academy of Sciences, Beijing, China

**Keywords:** gene loss, homologous recombination, DR/IR evolution, *Selaginella*, substitution rate, time divergence

## Abstract

Both direct repeats (DR) and inverted repeats (IR) are documented in the published plastomes of *Selaginella* species indicating the unusual and diverse plastome structure in the family Selaginellaceae. In this study, we newly sequenced complete plastomes of seven species from five main lineages of Selaginellaceae and also resequenced three species (*Selaginella tamariscina*, *Selaginella uncinata*, and *Selaginella moellendorffii*) to explore the evolutionary trajectory of Selaginellaceae plastomes. Our results showed that the plastomes of Selaginellaceae vary remarkably in size, gene contents, gene order, and GC contents. Notably, both DR and IR structures existed in the plastomes of Selaginellaceae with DR structure being an ancestral state. The occurrence of DR structure was at ∼257 Ma and remained in most subgenera of Selaginellaceae, whereas IR structure only reoccurred in *Selaginella* sect. *Lepidophyllae* (∼143 Ma) and *Selaginella* subg. *Heterostachys* (∼19 Ma). The presence of a pair of large repeats *psb*K-*trn*Q, together with DR/IR region in *Selaginella bisulcata*, *Selaginella pennata*, *S. uncinata*, and *Selaginella hainanensis*, could frequently mediate diverse homologous recombination and create approximately equal stoichiometric isomers (IR/DR-coexisting) and subgenomes. High proportion of repeats is presumably responsible for the dynamic IR/DR-coexisting plastomes, which possess a lower synonymous substitution rate (d*S*) compared with DR-possessing and IR-possessing plastomes. We propose that the occurrence of DR structure, together with few repeats, is possibly selected to keep the stability of plastomes and the IR/DR-coexisting plastomes also reached an equilibrium in plastome organization through highly efficient homologous recombination to maintain stability.

## Introduction

Plastid genomes (plastomes) of almost all land plants are highly conserved and present the canonical quadripartite structure with a pair of large inverted repeats (termed IR_A_ and IR_B_) separated by two single-copy regions (termed LSC and SSC) (Mower and Vickrey 2018). Normally, the range of the IR varies through expansion or contraction. Complete loss of the IR is rare but has been observed in some species of Fabaceae (Lavin et al. 1990; Cai et al. 2008), Geraniaceae (Guisinger et al. 2011; Blazier et al. 2016; Ruhlman et al. 2017), and Cactaceae (Sanderson et al. 2015). Remarkably, plastomes with a pair of large direct repeats (termed DR_A_ and DR_B_) have been documented for four species of Selaginellaceae, *Selaginella tamariscina* (Xu et al. 2018), *Selaginellavardei*, *Selaginellaindica* (Zhang et al. 2018), and *Selaginellakraussiana* (Mower et al. 2018) in land plants. The DR structure in Selaginellaceae was explained to have occurred by ∼50-kb fragment inversion with a complete IR_B_ being included, compared with the plastome of its sister family Isoetaceae (Mower et al. 2018; Zhang et al. 2018).

In addition to the exceptional existence of plastomes with DR structure, a salient fraction of land plants plastomes experienced significant structural rearrangements, with evidence of large inversions and loss of entire gene family, despite the overall conservation in structures and gene order (Mower and Vickrey 2018). A 30-kb inversion (*ycf*2-*psbM*) was detected in the large single copy (LSC) of ferns and seed plants plastomes relative to bryophytes and lycophytes, which is a strong evidence showing lycophytes are located as sister group of other vascular plants (Raubeson and Jansen 1992). The plastomes of ferns underwent two hypothetical inversions (CE inversion [*trnC* to *trnE*] and DE inversion [*trnD*-*trnY*]) within *rpoB*-*psbZ* (BZ) region from the ancestral gene order in eusporangiates to the derived gene order in core leptosporangiates whereas the plastome structures within these two groups were generally consistent, respectively (Gao et al. 2011, 2013; Grewe et al. 2013). Many other rearrangements also exist in some conifers (Chaw et al. 2018) and several angiosperm lineages like Campanulaceae, Fabaceae, and Geraniaceae (Mower and Vickrey 2018; Ruhlman and Jansen 2018). Inversion facilitated by recombination, transposition, and expansion/contraction of the IR has been suggested as three different mechanisms that cause rearrangements in land plants (Jansen and Ruhlman 2012). However, it is recently recognized that most plastomes exists as linear/concatemeric/highly branched complex molecules in plants and these rearrangement events are reinterpreted as result of a BIR (break-induced replication) -like, recombination-dependent replication mechanism between different linear plastome templates (Oldenburg and Bendich 2015). Furthermore, four families of nuclear-encoded proteins (MutS homolog 1 [*MSH*1], RecA-like recombinases, the organellar ssDNA-binding proteins [*OSB*s], and the Whirlies) have been characterized to target to both mitochondria and plastid, or some protein members of the four families target to only plastids and function as recombination surveillance machinery in plant plastids (Maréchal and Brisson 2010).

Gene and intron contents are highly conserved among the vast majority of land plants plastomes; however, numerous examples of gene loss or pseudogenization have been identified in several angiosperm lineages (Ruhlman and Jansen 2014). For example, most or all of the suite of 11 functionally related *ndh* genes have been lost independently in a small assortment of taxa with diverse habitat, including the parasitic *Epifagus* (Wicke et al. 2011), the mycoheterotrophic *Rhizanthella gardneri* (Delannoy et al. 2011), some members of the carnivorous Lentibulariaceae (Wicke et al. 2014), the xerophytic *Suguaro cactus* (Sanderson et al. 2015) and gnetales (Braukmann et al. 2009), the aquatic *Najas flexilis* (Peredo et al. 2013), and some taxa with less unusual life histories, such as Pinaceae (Braukmann et al. 2009) and *Erodium* (Blazier et al. 2011). In addition, the loss of protein coding genes and tRNA genes has occurred sporadically in different land plants lineages (Ruhlman and Jansen 2014; Wu and Chaw 2015). The fatty acid synthesis-related gene, *accD*, has been lost from plastomes of angiosperm at least seven times (Jansen et al. 2007). Similarly, more than a dozen parallel losses of ribosomal protein (*rps*/*rpl*) gene have occurred in different lineages of land plants (Ruhlman and Jansen 2014). Three major pathways of gene loss have been detected in land plants: 1) gene transfer to the nucleus (*infA*, *rpl*22, *rpl*32, and *accD*), 2) substitution by a nuclear-encoded, mitochondrial targeted gene product (*rps*16), and 3) substitution by a nuclear-encoded protein for a plastid gene product (*accD*, *rpl*23) (Jansen and Ruhlman 2012). The multiple independent *ndh* gene loss in different lineages is supposed to belong to the third pathway (Ruhlman et al. 2015).

Selaginellaceae, one of the most ancient vascular plants with nearly 400 Myr of evolutionary history (Banks 2009) is the largest family of lycophytes with ∼750 species classified into the only genus *Selaginella* (Jermy 1990; Weststrand and Korall 2016b; Zhou et al. 2016). *Selaginella* species have highly diverse growth forms, including creeping, climbing, prostrate, erect, and rosetted forms, and also inhabit an impressive range of habitats, from tropical rain forests to deserts, alpine, and arctic habitats (Zhang et al. 2013). Both mitochondrial and plastid genomes are more frequently subject to alterations under specific environmental conditions (Maréchal and Brisson 2010). With such a high diversity in habitat and growth forms and extremely long evolutionary history, complex plastomes with different structures are inferred in Selaginellaceae (Tsuji et al. 2007). However, only seven species of *Selaginella*, viz., *Selaginellauncinata* (Tsuji et al. 2007), *Selaginellamoellendorffii* (Smith 2009), *S. tamariscina* (Xu et al. 2018), *S. vardei*, *S. indica* (Zhang et al. 2018), *S. kraussiana*, and *Selaginellalepidophylla* (Mower et al. 2018) have been reported for their plastomes. Compared with the species from Lycopodiaceae and Isoetaceae of lycophytes (Wolf et al. 2005; Karol et al. 2010; Guo et al. 2016; Mower et al. 2018; Zhang et al. 2018), plastomes of *Selaginella* are, indeed, far less conserved in both structures and gene contents. Both *S. uncinata* and *S. moellendorffii* belong to subg. *Stachygynandrum* based on both morphology-based classification (Jermy 1986) and a recent molecular-based classification (Weststrand and Korall 2016b). However, their plastomes show divergent variation in structure. Several rearrangements, such as a 20-kb fragment inversion, a 17-kb fragment transposition and gene duplications, exist in these two species (Smith 2009). *Selaginella kraussiana*, belonging to subg. *Gymnogynum* (sensu Weststrand and Korall 2016a), is morphologically similar with S. uncinata, whereas the morphology of *S. tamariscina*, belonging to subg. *Stachygynandrum*, *S. lepidophylla*, belonging to subg. *Lepidophyllae* (sensu Weststrand and Korall 2016a), *S. vardei*, and *S. indica*, belonging to subg. *Rupestrae* (sensu Weststrand and Korall 2016a, 2016b) are also quite divergent from *S. uncinata* and *S. moellendorffii* in *S. tamariscina* and *S. lepidophylla* having rosetted plant and *S. vardei*, *S. indica* having helically arranged trophophylls, respectively. These four species both grow in extremely xeric habitat. Plastomes with DR rather than IR have been characterized in *S. tamariscina* (Xu et al. 2018), *S. indica, S. vardei* (Zhang et al. 2018), and *S. kraussiana* (Mower et al. 2018). Some other features of these plastomes are also quite distinctive. Only 6–12 different tRNA genes remain in *Selaginella*, and GC content in *Selaginella* plastomes is significantly higher (51–54.8%) than the plastomes of other land plants (<43%) (Tsuji et al. 2007; Smith 2009). Such extensive rearrangement events and extraordinary gene content have never been reported in other lycophytes and fern families (Karol et al. 2010; Guo et al. 2016; Mower and Vickrey 2018).

The divergent variation in structure and gene content exhibited by Selaginellaceae plastomes make it an ideal family to study the complexity and diversity of plastomes. Furthermore, the extent of genomic change in other lineages of *Selaginella* species has not been fully investigated. Therefore, we sequenced a total of ten plastomes from species belonging to six different main lineages of Selaginellaceae using next generation sequencing method and combined with previously published plastomes of lycophytes to reach the following goals: 1) document plastome characteristics from major lineages of Selaginellaceae, 2) explore the evolutionary trajectory and dynamic conformations of DR/IR structure in plastomes of Selaginellaceae, and 3) reveal the potential correlations among plastome rearrangements, substitution rate, and number of repeats.

## Materials and Methods

### Taxon Sampling

Seven taxa (*Selaginellalyallii*, *Selaginellaremotifolia*, *Selaginellasanguinolenta*, *Selaginelladoederleinii*, *Selaginellapennata*, *Selaginellabisulcata*, and *Selaginellahainanensi*s) from five main lineages of Selaginellaceae representing four subgenera of Zhou and Zhang (2015) and three subgenera of Weststrand and Korall (2016a) were sampled (supplementary table S1, Supplementary Material online). The previously published plastomes of *S. tamariscina*, *S. moellendorffii*, and *S. uncinata* were resequenced to confirm their structures. Previously published plastomes of *S. vardei*, S*. indica* (Zhang et al. 2018), *S. kraussiana*, and *S. lepidophylla* (Mower et al. 2018) were also included. Outgroups include three species from Isoetaceae and five species from Lycopodiaceae (Wolf et al. 2005; Karol et al. 2010; Guo et al. 2016; Mower et al. 2018; Schafran et al. 2018) as references. We followed the classification of Zhou and Zhang (2015) to describe the lineages represented by our species.

### DNA Extraction, Sequencing, and Assembly

The total genomic DNAs were isolated from silica-dried materials with a modified cetyl trimethylammonium bromide (CTAB) method (Li et al. 2013). Library construction was performed with NEBNext DNA Library Prep Kit (New England Biolabs, Ipswich, MA) and sequencing was finished by Illumina HiSeq 2500 (Illumina, San Diego, CA). Illumina paired-end reads of each species were mapped to *S. uncinata* (AB197035) (Tsuji et al. 2007) and *S. moellendorffii* (FJ755183) (Smith 2009) with medium to low sensitivity in five to ten iterations in Geneious v. 9.1.4 (Kearse et al. 2012) (Biomatters, Inc., Auckland, New Zealand; https://www.geneious.com; last accessed February 28, 2019). The mapped reads were then assembled into contigs in Geneious. We also used bandage v. 0.8.1 (Wick et al. 2015), a program for visualizing de novo assembly graphs, to help select contigs of plastome and analyze de novo assemblies by importing the fastg file created by the recent developed pipeline GetOrganelle (Jin et al. 2018). The contigs obtained from both ways were then combined and imported into Geneious to extend and assemble into the complete plastomes.

### Gene Annotation

Gene annotations were performed with local BLAST (Delsuc et al. 2005) using the plastomes of *Huperziaserrata* (Guo et al. 2016), *Isoetesflaccida* (Karol et al. 2010), *S. uncinata* (Tsuji et al. 2007), and *S. moellendorffii* (Smith 2009) as references. Putative start and stop codons were defined based on similarity with known sequences. The tRNAs were further verified using tRNAscan-SE version 1.21 (Lowe and Eddy 1997; Schattner et al. 2005) and ARAGORN (Laslett and Canback 2004). Circular and linear plastome maps were drawn in OGDraw version 1.2 (Lohse et al. 2007).

### PCR Confirmation of the Plastome Structure of Representative Species in Four Subgenera

We selected 18 representatives (supplementary table S2, Supplementary Material online) from four subgenera to confirm whether the DR structure is ubiquitous in plastomes of three subgenera and IR structure only exists in one subgenus. Primers were designed at the flanking regions of rearrangement end points and DR/IR region boundaries. Considering the distant relationship among these subgenera, we designed the primers (supplementary table S3, Supplementary Material online) for each subgenus, respectively. The PCR amplifications were performed in a total volume of 25 μl containing 2.5 μl of 10× Ex Taq Buffer, 2.5 μl of dNTP Mixture (2.5 mM each), 2 μl of each primer (5 mM), 0.15 μl of TaKaRa Ex Taq (5 units/µl), and 20 ng of template DNA. Cycling conditions were 94 °C for 3 min, followed by 35 cycles of 94 °C for 30 s, 52 °C for 1 min, and 72 °C for 1.5 min, and a final extension of 72 °C for 10 min. The PCR products were verified by electrophoresis in 1% agarose gels stained with ethidium bromide and sequenced by the Company of Majorbio, Beijing, China.

### Plastome Rearrangement Analyses

In order to identify the putative presence of large structural variation within the *Selaginella* plastomes, whole genome alignment among 16 lycophyte species (14 Selaginellaceae species, *I. flaccida*, and *H. serrata*) was performed using the progressiveMauve algorithm in Mauve v 2.3.1 (Darling et al. 2010). A copy of DR/IR was removed from the plastomes. The Locally colinear blocks (LCBs) identified by the Mauve alignment were numbered to estimate the genome rearrangement distances (supplementary table S4, Supplementary Material online). Genes in each block were also listed (supplementary table S5, Supplementary Material online). Two types of genome rearrangement distances, break point (BP) and inversions (IVs), were calculated using the web server of the Common Interval Rearrangement Explore (CREx) (Bernt 2007) using the conserved plastome of *H. serrata* as a reference.

### Repeat Analyses

Repeats within the 16 lycophytes plastomes (14 Selaginellaceae species, *I. flaccida*, and *H. serrata*) were identified by RepeatsFinder (Volfovsky et al. 2001) with default parameters (repeat size >15 bp). One copy of the DR/IR was removed from all plastomes used. The circular layouts of repeats in our newly sequenced plastomes were visualized using the *circlize* package (Gu et al. 2014) in R. Furthermore, the correlation between the number of repeats and the degree of genome rearrangements, BPs, and IVs distance were tested using Pearson test in R v. 3.4.1 (R Development Core Team 2012). In addition, the correlation analysis following phylogenetically independent contrasts (PICs) method (Felsenstein 1985) was carried out to exclude the possible phylogeny signal using ape package in R v. 3.4.1. The phylogenetic tree was adopted with ML tree in section “Phylogenetic Analysis, Ancestral States Reconstruction, and Divergence Time Estimation.”

### Nucleotide Substitution Rate Analyses

Forty-six protein-coding genes (supplementary table S6, Supplementary Material online) from single-copy regions of 14 Selaginellaceae species and 1 outgroup, *I. flaccida*, were extracted from the plastomes and aligned at the protein level by MAFFT (Katoh and Standley 2013) using the translation-aligned function in Geneious v. 9.1.4 (Kearse et al. 2012). Poorly aligned regions were removed by using Gblocks v. 0.91b (Castresana 2000) with default parameters. The data set for substitution rate comparison between plastomes with DR, IR, and IR/DR-coexisting structure in Selaginellaceae includes all 14 *Selaginella* species. The data set for comparison of genes inside or outside the ∼50-kb inversion (supplementary table S6, Supplementary Material online) includes nine *Selaginella* species with plastomes of DR structure. The pairwise synonymous substitution rate (d*S*), nonsynonymous substitution rate (d*N*), and d*N*/d*S* of each individual gene was estimated using PAML v. 4.9 (run mode=−2) (Yang 2007) with codon frequencies determined by the F3 × 4 model. The significance of differences of d*S*, d*N*, and d*N*/d*S* was assessed using Wilcoxon rank sum tests in R v. 3.4.1 (R Development Core Team 2012).

### Phylogenetic Analysis, Ancestral States Reconstruction, and Divergence Time Estimation

Forty-six protein-coding genes (supplementary table S6, Supplementary Material online) with 30,741 bases shared by 22 lycophyte species (14 Selaginellaceae, 3 Isoetaceae, and 5 Lycopodiaceae) were extracted and aligned using Multiple Alignment in Geneious v. 9.1.4 (Kearse et al. 2012) under the automatic model selection option with some manual adjustments. The first and second sites of each codon were selected in MEGA 7.0 (Tamura et al. 2007) to eliminate the effect of third site base substitution saturation. Phylogenetic analysis was performed using maximum likelihood methods on the RAxML web server with 1,000 bootstrap replicates and the GTR+G model was selected based on Akaike information criterion (AIC) in jModeltest 2.1.7 (Darriba et al. 2012).

The evolution of DR and IR structures was reconstructed with likelihood method implemented in Mesquite v. 2.7.5 (Maddison and Maddison 2011). The character state was coded as IR and DR for each specie, and the character was treated as unordered and equally weighted. The character was plotted onto 1,000 trees that were sampled in the ML analyses and the results were finally summarized as percentage of changes of character states on a given branch among all 1,000 trees.

**Fig. 1.**
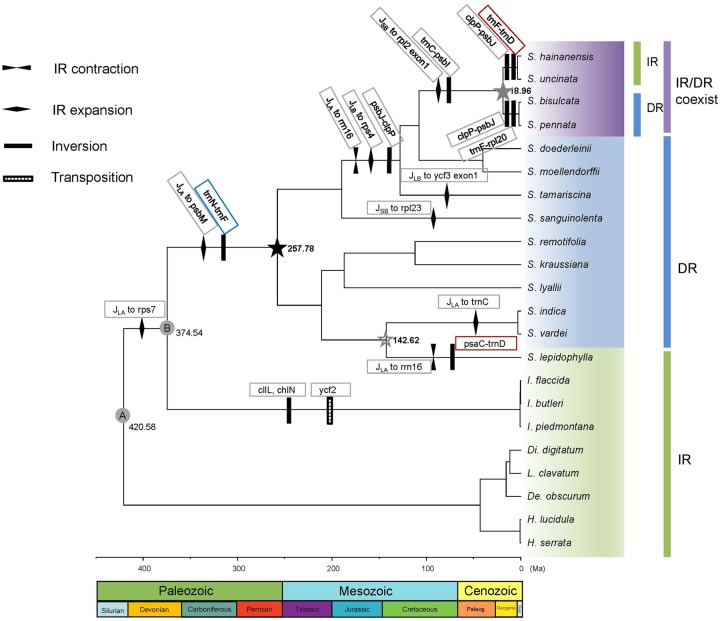
—Phylogeny reconstruction and time divergence estimation of lycophytes with plastome rearrangement events mapped on the branches. Node A–B represent the calibration nodes. Node A: fossil calibration of the root age corresponding to the split of Lycopodiopsida and Isoetopsida (392–451 Ma); node B: fossil node separating Selaginellaceae and its sister family Isoetaceae (372–392 Ma). Black star represents the occurrence time of plastomes with DR structure, gray star represents the occurrence time of plastomes with IR/DR-coexisting structure and hollow gray star represents the occurrence time of plastomes with IR structure.

The divergence time of DR/IR occurrence was estimated using BEAST version 1.8.2 (Suchard et al. 2018) with two fossil calibration nodes employed. A fossil calibration of the root age corresponded to the split of Lycopodiopsida and Isoetopsida (fig. 1, node A: [392–451 Ma]) (Morris et al. 2018) with a selection of normal distribution as a prior. The other fossil calibration of the node separated Isoetaceae and Selaginellaceae (fig. 1, node B: [372–392 Ma]) (Kenrick and Crane 1997) with a lognormal distribution as a prior. A relaxed clock with lognormal distribution of uncorrelated rate variation was specified, and a birth–death speciation process with a random starting tree was adopted. The MCMC chain was run for 500 million generations, sampled every 1,000 generations. The effective sample size (ESS) was checked in Tracer v 1.5 (Rambaut and Drummond 2009). The maximum clade credibility tree was generated using TreeAnnotator in BEAST and the tree was plotted using FigTree v. 1.4.3 (Rambaut 2017). The events of DR/IR origins, rearrangements, and DR/IR expansion/contraction in Selaginellaceae were mapped on the phylogenetic tree to explore the evolutionary trajectory of Selaginellaceae plastomes.

## Results

### Characteristics of Selaginellaceae Plastomes

**Fig. 2.**
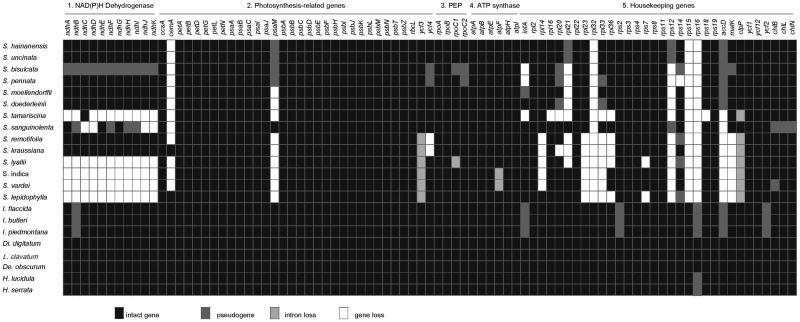
—Protein-coding genes in Selaginellaceae and other lycophytes. Intact genes per species are indicated by black boxes, dark gray box represent putative pseudogenes, light gray and white boxes mark intron and gene losses, respectively. PEP, plastid-encoded RNA polymerase. note.—The taxa in the figure is sorted based on the phylogeny of figure 1.

The general features of 14 Selaginellaceae plastomes and 7 other lycophytes are summarized in table 1 and supplementary table S1, Supplementary Material online. Compared with other lycophytes, the plastomes of Selaginellaceae showed remarkable variation in size, ranging from roughly 110 kb in *S. lyallii* to 147 kb in *S. sanguinolenta*. Size variability was partly due to the DR/IR regions, which expanded to ∼16 kb in *S. sanguinolenta* and reduced to ∼7 kb in *S. lepidophylla*. Gene content was also variable in Selaginellaceae plastomes due to a number of gene losses (table 1 and fig. 2). Lycophyte plastomes generally contained 120 different genes (86 protein-coding genes, 30 tRNA genes, and 4 *rrn* genes), whereas it ranged from 73 different genes (61 protein-coding genes, 8 tRNA genes, and 4 *rrn* genes) in *S. tamariscina* to 102 different genes (76 protein-coding genes, 22 tRNA genes, and 4 *rrn* genes) in *S. sanguinolenta*. Intron loss was detected in *atpF*, *clpP*, *rpo*C1, and *ycf*3 genes. The GC content in Selaginellaceae plastomes was significantly higher than those in Isoetaceae and Lycopodiaceae. The average GC content was 52.9% in Selaginellaceae (ranging from 50.7% in *S. lyallii* to 56.5% in *S. remotifolia*) and 36.7% in other lycophytes (table 1).

**Table 1:** Plastome Characteristics for Representative Selaginellaceae in Comparison to Other Lycophytes

Species	Size (bp)	LSC (bp)	SSC (bp)	IR (bp)	No. Different Genes	No. Different Protein-Coding Genes (duplicated)	No. Different tRNA Genes (duplicated)	No. Different rRNA Genes (duplicated)	GC Content (%)
*Selaginella uncinata*	144,161	77,752	40,851	12,779	97	81 (3)	12 (3)	4 (4)	54.9
*S. hainanensis*	144,201	77,780	40,819	12,801	97	81 (3)	12 (3)	4 (4)	54.8
*S. bisulcata*	140,509	55,598	59,659	12,626	94	78 (3)	12 (3)	4 (4)	52.8
*S. pennata*	138,024	54,979	59,847	11,599	90	77 (3)	9 (2)	4 (4)	52.9
*S. moellendorffii*	143,525	58,198	61,129	12,099	96	78 (1)	14 (2)	4 (4)	51
*S. doederleinii*	142,752	57,841	62,865	11,023	96	78 (1)	14 (2)	4 (4)	51.1
*S. tamariscina*	126,700	53,299	47,741	12,830	73	61 (1)	8 (1)	4 (4)	54.1
*S. sanguinolenta*	147,148	54,436	59,650	16,531	102	76 (4)	22 (3)	4 (4)	50.8
*S. remotifolia*	131,866	46,351	55,851	14,832	87	71 (3)	12 (2)	4 (4)	56.5
*S. kraussiana*	129,971	46,049	54,728	14,597	85	71 (3)	10 (2)	4 (4)	52.3
*S. lyallii*	110,411	44,943	45,276	10,096	76	60 (1)	13 (2)	4 (4)	50.7
*S. indica*	122,460	45,711	48,395	14,177	78	62 (3)	12(3)	4 (4)	53.6
*S. vardei*	121,254	45,792	47,676	13,893	78	62 (3)	12(3)	4 (4)	53.2
*S. lepidophylla*	114,693	80,625	19,452	7,308	80	64 (0)	12 (1)	4 (4)	51.9
*Isoetes flaccida*	145,303	91,862	27,275	13,118	121	85 (3)	32 (5)	4 (4)	37.9
*Isoetes butleri*	144,912	91,543	27,171	13,099	121	85 (2)	32 (5)	4 (4)	38.0
*Isoetes piedmontana*	145,030	91,748	27,198	13,042	121	85 (2)	32 (5)	4 (4)	38.0
*Diphasiastrum digitatum*	159,614	106,400	19,444	16,885	122	87 (1)	31 (5)	4 (4)	35.7
*Lycopodium clavatum*	151,819	105,643	21,342	12,417	122	87 (0)	31 (5)	4 (4)	34.5
*Dendrolycopodium obscurum*	160,877	19,465	105,928	17,742	122	87 (1)	31 (5)	4 (4)	35.0
*Huperzia lucidula*	154,373	104,088	19,657	15,314	121	86 (2)	31 (5)	4 (4)	36.2
*Huperzia serrata*	154,176	104,080	19,658	12,219	121	86 (2)	31 (5)	4 (4)	36.3

A noticeable plastome structure with DR was documented in *S. tamariscina* (Xu et al. 2018), *S. indica*, *S. vardei* (Zhang et al. 2018), and *S. kraussiana* (Mower et al. 2018). With our newly selected species being sequenced, we assembled the plastomes of seven species (*S. lyallii*, *S. remotifolia*, *S. sanguinolenta*, *S. doederleinii*, *S. moellendorffii*, *S. bisulcata*, and *S. pennata*) into the DR structure (fig. 1). Besides, we found that the length of two single-copy regions (LSC and SSC) changed into almost equal size. However, the length of LSC (44.9–57.8 kb) was slightly shorter than that of SSC (45.3–62.8 kb) (table 1), which mainly resulted from a relocation of ∼35-kb fragments from LSC to SSC region. Only the plastomes of *S. hainanensis* and *S. uncinata* were assembled into the typical IR structure (fig. 1). Besides, the recent published plastome of *S. lepidophylla* also possess IR structure. We consider the assembled plastome structures as master forms in the following analyses.

### Confirmation of DR/IR Structures in Plastomes of Representative Species

The DR structure in plastome of *S. vardei* for subg. *Rupestrae sensu*Weststrand and Korall (2016a) (*Selaginella* sect. *Homoeophyllae sensu*Zhou and Zhang 2015) have been confirmed by Zhang et al. (2018). The PCR confirmation of 18 representative species with different structure (supplementary table S2, Supplementary Material online) from four subgenera *sensu*Zhou and Zhang (2015) suggested that DR structure is ubiquitous in subg. *Stachygynandrum*, subg. *Pulviniella*, subg. *Ericetorum*, and subg. *Boreoselaginella* (supplementary fig. S2*b*–*d*, Supplementary Material online). Particularly, the resequenced plastome of *S. moellendorffii* was also found to possess DR structure, which is inconsistent with the previously published IR structure (Smith 2009). The PCR confirmation of nine species from the same subgenus as *S. moellendorffii* further supported the DR structure (supplementary fig. S2*b*, Supplementary Material online). Therefore, we used the resequenced DR-possessing plastome of *S. moellendorffii* for the following analyses. For subg. *Heterostachys*, the PCR confirmation of five species indicated that the IR structure only existed in the sect. *Oligomacrosporangiatae* which *S. uncinata* and *S. hainanensis* were located (supplementary fig. S2*a*, Supplementary Material online).

### tRNA Gene Loss

**Fig. 3.**
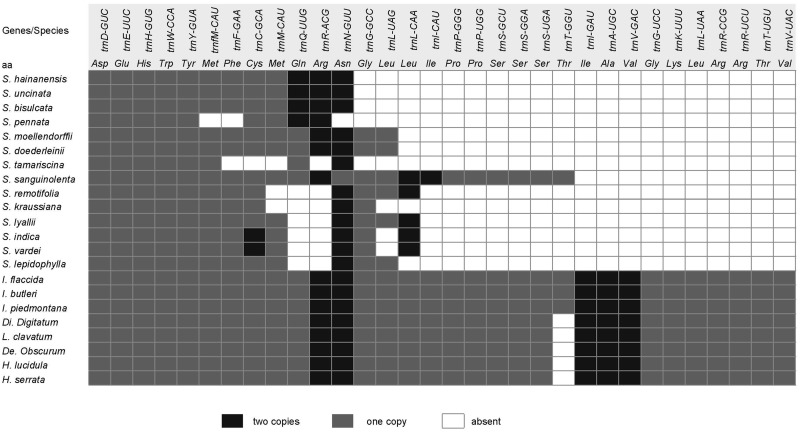
—tRNA genes in Selaginellaceae and other lycophytes. Black box represents tRNA genes with two copies, gray box represents tRNA genes with one copy, and white box represents tRNA gene loss. note.—The taxa in the figure is sorted based on the phylogeny of figure 1.

The most noticeable feature of gene content is the tRNA gene loss (table 1 and fig. 3). Plastomes in other land plants usually contain 30 different tRNA genes whereas Selaginellaceae plastomes have experienced an extensive tRNA gene loss and varies greatly in different lineages. Twenty-two different tRNA genes were annotated in plastome of *S. sanguinolenta*, whereas only eight different tRNA genes existed in *S. tamariscina* plastome. Some vestiges of tRNA genes (e.g., *trnA-UGC* exon2 and *trnI-GAU* exon1 between *rrn*16 and *rrn*23, *trnV-UAC* exon1 between *trnM-CAU* and *trnF-GAA*) were observed in plastome of *S. sanguinolenta*.

### Protein-Coding Gene/Intron Loss

The loss and putative pseudogenization (with premature stop codons) of protein-coding genes in Selaginellaceae plastomes are shown in figure 2, mainly focusing on NAD(P)H-dehydrogenase complex-encoding genes (*ndh* genes) and ribosomal protein-encoding genes (*rpl*/*rps* genes). In *Selaginella*, the gene loss or pseudogenization of *ndh* genes occurred to a different extent. All *ndh* genes were lost in *S. vardei*, *S. indica*, *S. lyallii*, and *S. lepidophylla* and only one functional *ndhC* remained in *S. tamariscina*, whereas in *S. sanguinolenta*, four *ndh* genes (B, F, H, I) became pseudogenes, four (C, D, J, K) were completely lost, and other three (A, E, G) were still functional. The whole set of n*dh* genes in *S. bisulcata* were all pseudogenized because of premature stop codons caused by reading frame shift, whereas they were intact and functional in its sister species, *S. pennata*. In addition to the whole set of gene loss in specific species, several genes were lost across the whole family. The genes of *cemA*, *rpl*32, *rps*15, and *rps*16 were absent in plastomes of almost all *Selaginella* species except the *S. kraussiana* and *S. lepidophylla*, but present in all plastomes of outgroups. The gene *accD* was nonfunctional (pseudogenized or lost) in all Selaginellaceae and Isoetaceae, but was functional in Lycopodiaceae. Besides, a number of ribosomal genes (*rpl*14, 16, 20, 21, 23, 33, 36 and *rps*12, 14, 18) were lost or pseudogenized independently in different lineages of Selaginellaceae. Although *infA* is present in all bryophyte and fern lineages, it is pseudogenized in the plastomes of Isoetaceae and *S. moellendorffii* and lost in three *Selaginella* species (*S. bisulcata*, *S. pennata*, and *S. tamariscina*). *matK* gene was absent in *S. remotifolia*, *S. kraussiana*, *S. lyallii*, *S. indica*, *S. vardei*, and *S. lepidophylla*, and pseudogenized in *S. bisulcata*. *rpoC*1 and *rpoC*2 were pseudogenized in *S. bisulcata* and *rpoC*1 was pseudogenized in *S. pennata*. All three *chl* genes were pseudogenized in *S. sanguinolenta*, and only *chlB* was pseudogenized in *S. vardei*. However, they were all intact in other *Selaginella* species. The gene *psaM* was nonfunctional (pseudogenized or lost) in all *Selaginella* species except *S. sanguinolenta*, which possessed an intact *psaM* gene. Another event worth noticing was the intron loss in Selaginellaceae. Two introns remained in *clpP* of *S. doederleinii* and *S. sanguinolenta*, none in *S. tamariscina*, *S. remotifolia*, *S. kraussiana*, *S. lyallii*, *S. indica*, *S. vardei*, and *S. lepidophylla*, and one remained in other species of *Selaginella*. The intron of *atpF* gene was lost in *S. indica* and *S. vardei*, the intron of *rpoC1* was lost in *S. lyallii*, and the intron 2 of *ycf*3 gene was lost in *S. vardei*, *S. indica*, *S. lepidophylla*, *S. lyallii*, *S. kraussiana*, and *S. remotifolia*.

### Rearrangement Events in Plastomes of Selaginellaceae

Twenty locally collinear blocks (LCBs) (supplementary table S5, Supplementary Material online) shared by Selaginellaceae and outgroups were identified by Mauve whole plastome alignment. Each LCB for 14 plastomes was numbered from 1 to 20 and assigned a ± showing strand orientation (supplementary table S4, Supplementary Material online). The order of the 20 LCBs in each plastome was number coded for estimating breakpoint (BP) and inversions (IVs) distances referring to the plastome organization of *H. serrata*. The pairwise comparison of the two types of plastome rearrangement distances is shown in table 2. The two distances were highly correlated (*P* < 0.001, *r* = 0.977). Both distances were used as the estimation of the degree of genome rearrangement in the later analyses.

**Table 2:** Pairwise Comparison of Genome Rearrangement Distances

	Hs	If	Sle	Sv	Si	Sr	Sk	Sly	Ss	St	Sd	Sm	Sp	Sb	Sh
*Huperzia serrata*	—														
*Isoetes flaccida*	8/6	—													
*Selaginella lepidophylla*	4/2	10/8	—												
*S. vardei*	5/3	10/9	5/3	—											
*S. indica*	5/3	10/10	5/3	0	—										
*S. remotifolia*	5/3	10/11	5/3	3/3	3/3	—									
*S. kraussiana*	5/3	10/12	5/3	3/3	3/3	0/0	—								
*S. lyallii*	5/3	10/13	5/3	3/3	3/3	0/0	0/0	—							
*S. sanguinolenta*	5/3	10/14	5/3	3/3	3/3	0/0	0/0	0/0	—						
*S. tamariscina*	4/2	11/8	4/2	5/4	5/4	5/4	5/4	5/4	5/4	—					
*S. doederleinii*	4/2	11/8	4/2	5/4	5/4	5/4	5/4	5/4	5/4	0/0	—				
*S. moellendorffii*	4/2	11/8	4/2	5/4	5/4	5/4	5/4	5/4	5/4	0/0	0/0	—			
*S. pennata*	6/5	13/11	7/5	7/6	7/6	7/6	7/6	7/6	7/6	7/5	7/5	7/5	—		
*S. bisulcata*	6/5	13/11	7/5	7/6	7/6	7/6	7/6	7/6	7/6	7/5	7/5	7/5	0/0	—	
*S. hainanensis*	6/5	12/10	6/5	7/6	7/6	7/6	7/6	7/6	7/6	7/5	7/5	7/5	3/2	3/2	—

Note.—The lower diagonal refers to BP/IV distance. The taxa in the table is sorted based on the phylogeny of Selaginellaceae in this study.

The plastome rearrangement between *I. flaccida* and *S. vardei* was described in Zhang et al. (2018). The plastomes of *S. vardei*, *S. indica*, *S. lyallii*, *S. kraussiana*, *S. remotifolia*, and *S. sanguinolenta* were almost syntenic except the different extent loss of *ndh* genes (fig. 2 and supplementary fig. S1, Supplementary Material online) and slightly change of DR boundaries (fig. 1). However, the organization of *S. lepidophylla* plastome was quite exceptional with IR structure. An ∼68-kb inversion (*psaC*-*trnD*) was found when comparing plastomes of *S. lepidophylla* and *S. vardei*. Compared with the plastome of *S. sanguinolenta*, an inversion of 8-kb *psbJ*-*clpP* fragment (block −15, −14) was observed in the plastome of *S. tamariscina*. The *psbJ*-*clpP* inversion was also shared by the plastomes of *S. doederleinii* and *S. moellendorffii*, which are basically snytenic with that of *S. tamariscina*. The inversion of an ∼20-kb *trnC*-*psbI* fragment (block 4, 5), absent in *S. moellendorffii* and other species, showed a shared character in the plastomes of *S. bisulcata*, *S. pennata*, *S. hainanensis*, and *S. uncinata*. Two extra inversions existed when comparing the plastomes of *S. moellendorffii* and *S. bisulcata*. The first inversion of a 9-kb *psbJ*-*clpP* fragment (block 14, 15) was located at one end of the second inversion of 20-kb *rpl*20-*trnF* fragment (block −14, −13, −12) with *clpP* (block −15) being excluded. The plastome organization of *S. pennata* was basically syntenic with its sister species, *S. bisulcata*.

The plastomes of *S. bisulcata* and *S. pennata* were assembled into DR structure whereas the plastomes of both *S. uncinata* and *S. hainanensis*, which belong to the same subgenus, retained the typical IR structure as observed in other land plants. Therefore, how the DR region change back to IR again is an intriguing phenomenon. The comparison between plastomes of *S. moellendorffii* and *S. uncinata* displayed two more inversions except the aforementioned *trnC*-*psbI* inversion. The first inversion was an ∼65-kb fragment of *trnD*-*trnF* (block 9, 10, 11, −17, −16, −15, −14, −13, −12); followed by the inversion of an ∼9-kb fragment of *psbJ*-*clpP* (block 14, 15). The second inversion occurred inside of the first inversion. With one copy of repeat region (DR_B_) inside, the first inversion changed *S. uncinata* repeat regions into an IR structure. Since *S. hainanensis* is sister to *S. uncinata*, the plastome structure is basically syntenic with each other.

### Expansion and Contraction of DR/IR Regions in *Selaginella* Plastomes

Repeat regions of plastomes in different lineages of *Selaginella* ranged from 7,308 bp in *S. lepidophylla* to 16,531 bp in *S. sanguinolenta* (table 1). In the lineages of *S. vardei*, *S. indica*, *S. lyallii*, *S. kraussiana*, *S. remotifolia*, and *S. sanguinolenta*, one end of the DR_B_ region expanded and incorporated genes (*rps*7, *ndhB*, *psbM*, *petN*, and *trnC*) from LSC region to a different extant, whereas the other end of the DR_B_ region contracted to *trnN* or *trnR* (fig. 1 and supplementary fig. S1, Supplementary Material online). Part of gene *rpl*23 from SSC was also included into DR region in plastome of *S. sanguinolenta*. However, IR region in *S. lepidophylla* plastome contracted the most in Selaginellaceae containing no protein-coding genes. In the lineages containing *S. tamariscina*, *S. doederleinii*, *S. bisulcata*, and *S. hainanensis*, one end of the DR_A_ next to the LSC region contracted to *rrn*16 or *rpl*23 whereas the other end of the DR_A_ next to the SSC region expanded to include *rps*4 and even one exon of *ycf*3 (fig. 1 and supplementary fig. S1, Supplementary Material online).

### Repeats in *Selaginella* Plastomes

**Fig. 4.**
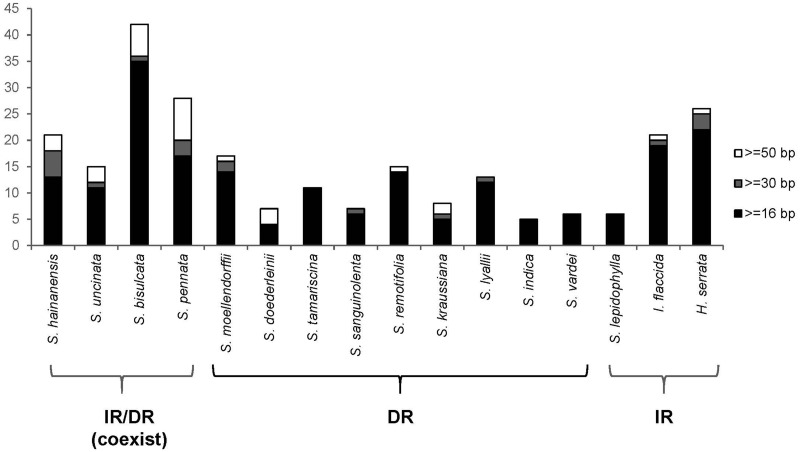
—Statistics of repeat analyses in lycophytes.

**Fig. 5.**
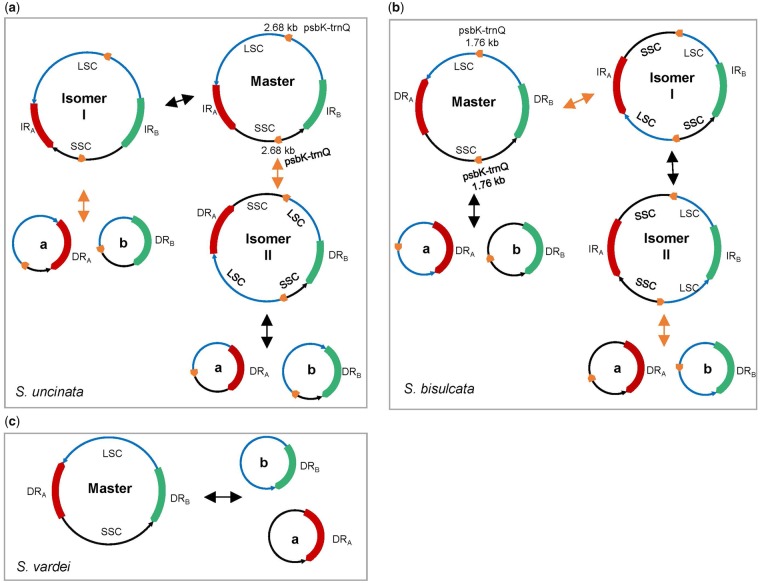
—Dynamic structures of plastomes with DR/IR structure of Selaginellaceae. (*a*) Recombination activities between repeats in plastomes of *Selaginella uncinata* and *S. hainanensis*. The red and green block represents two copies of IR with arrows at the end showing orientation and the short orange blocks represent two copies of repeats (*psb*K-*trn*Q). The black double arrows show recombination between IR and the orange double arrows show recombination between repeats *psb*K-*trn*Q; (*b*) recombination activities between repeats in plastomes of *S. bisulcata* and *S. pennata*. The red and green blocks represent two copies of DR with arrows at the end showing orientation and the short orange blocks represent two copies of repeats (*psb*K-*trn*Q). The black double arrows show recombination between DR and the orange double arrows show recombination between repeats *psb*K-*trn*Q; (*c*) recombination activities between repeats in plastomes with DR structure.

The total number of repeats in Selaginellaceae plastomes were slightly lower than that in Isoetaceae and Lycopodiaceae plastomes, whereas repeats with the size >50 bp were the most in Selaginellaceae plastomes (fig. 4 and supplementary fig. S5, Supplementary Material online). Among the 16 plastomes compared, *S. bisulcata* contained the most repeats (42) in lycophytes, with *S. pennata* possessing the most repeats >50 bp (8), whereas *S. indica* had the fewest (5) in total. The number of repeats varied among the species of Selaginellaceae, and the plastomes of *S. bisulcata* and *S. pennata* contained the most repeats (42 and 28) of all. Besides, 16 copies of a 17-bp repeat unit were dispersed in the intergenic region of *trnF*-*chlL* in plastome of *S. lepidophylla.* The 16 copies of repeat units can pair into a number of repeats; therefore, we did not display these repeats in figure 4. The degree of plastome rearrangement estimated by BP and IV distances were moderately correlated with the total number of repeats in all 14 *Selaginella* species (BP, *r* = 0.616, *P* = 0.019; IVs, *r* = 0.701, *P* = 0.005) (supplementary fig. S6*a* and *b*, Supplementary Material online). However, the results of PICs method showed that correlation between rearrangements distance and number of repeats was not supported (BP, *r* = 0.263, *P* = 0.186; IVs, *r* = 0.325, *P* = 0.137) (supplementary fig. S6*c* and *d*, Supplementary Material online). We did find some repeats, which are able to mediate homologous recombination, flanking several rearrangement end points. A pair of short repeats existed at the flanking region of ∼20-kb *psbI*-*trnC* inversion in *S. bisulcata* (48 bp) and *S. pennata* (60 bp) (supplementary fig. S5, Supplementary Material online), and another pair of repeats at the flanking region of 10-kb *psbJ*-*clpP* inversion in *S. doederleinii* (166 bp) and *S. moellendorffii* (264 bp) (supplementary fig. S5, Supplementary Material online) suggesting that repeats >50 bp may have facilitated rearrangements in Selaginellaceae. Besides, a pair of ∼1.8-kb IRs exists in plastomes of *S. bisulcata* and *S. pennata*, and a pair of ∼2.7-kb DRs exists in plastomes of *S. uncinata* and *S. hainanensis*. These two pairs of repeats, together with DR and IR, are hypothesized to be able to frequently mediate diverse homologous recombination, and create approximately equal stoichiometric subgenomes and isomers. Therefore, both IR and DR structures were inferred to exist dynamically (IR/DR coexist) in plastomes of *S. bisulcata*, *S. pennata*, *S. uncinata*, and *S. hainanensis* (fig. 5).

### Nucleotide Substitution in *Selaginella* Plastomes

**Fig. 6.**
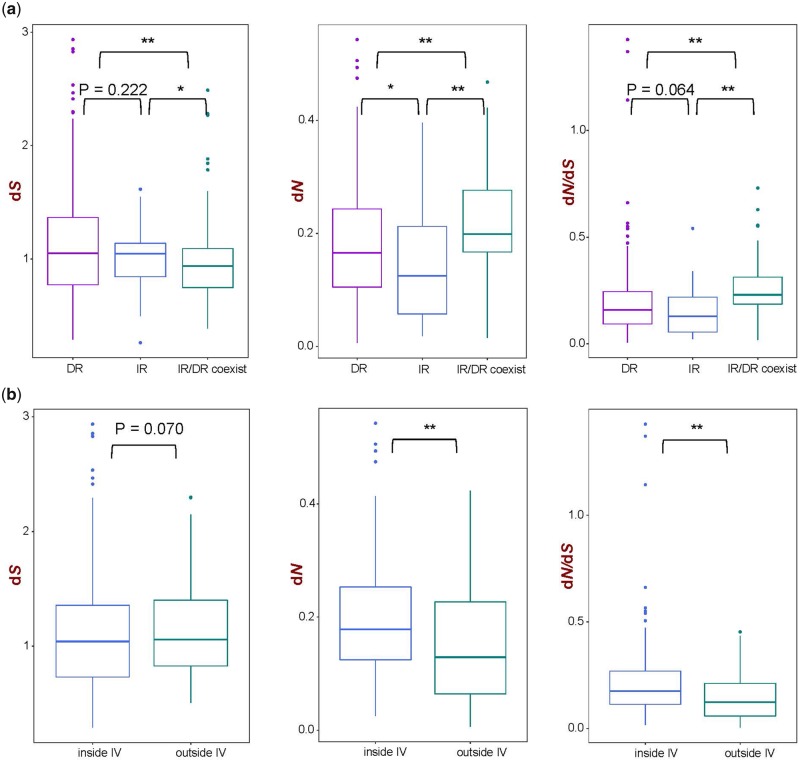
—Nucleotide substitution rate analyses in Selaginellaceae. (*a*) Substitution rate of genes from DR-possessing, IR-possessing, and IR/DR-coexisting plastomes and (*b*) substitution rate of genes inside IV and outside IV from DR-possessing plastomes.

The pairwise substitution rate comparison of 46 genes from single-copy regions of 14 *Selaginella* plastomes showed that the d*S* value for the genes of DR-possessing and IR-possessing plastomes had no significant difference (*P* = 0.222), and both d*S* value were significantly higher than those of IR/DR-coexisting plastomes (*P* < 0.01, *P* < 0.01). The d*N* and d*N*/d*S* value for DR-possessing plastomes was slightly higher than that of IR-possessing plastomes, whereas the significant higher value of d*N* and d*N*/d*S* was observed for the IR/DR-coexisting plastomes (*P* < 0.01, *P* < 0.01) (fig. 6*a*). Of the 46 genes, 28 genes were inside the ∼50-kb inversion and 18 genes were outside the inversion for DR-possessing plastomes. Comparison between genes inside and outside IV showed that d*S* was slightly lower in genes inside IV than outside IV without significant difference (*P* = 0.070), whereas the opposite trend was observed for d*N* and d*N*/d*S* (*P* < 0.01, *P* < 0.01) (fig. 6*b*).

### Phylogenetic Reconstruction and Divergence Time Estimation

**Fig. 7.**
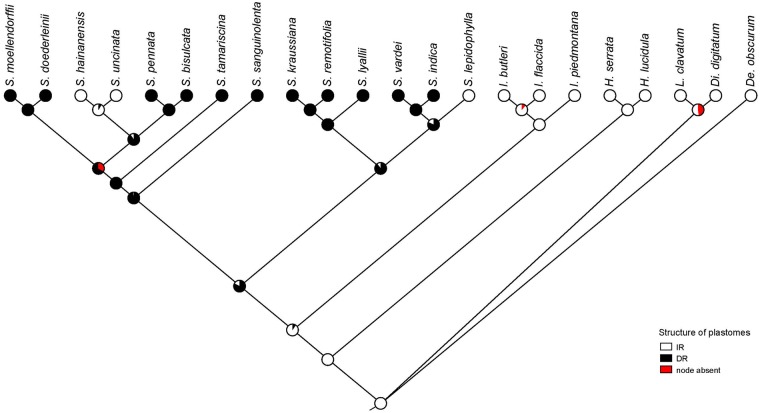
—Ancestral states reconstruction of Selaginellaceae plastomes structure reconstructed with maximum likelihood over 1,000 trees obtained in the ML analysis of the plastome data set. Pie charts show the percentage of node absence in the input 1,000 trees and the average likelihood received by each state across all input trees possessing that node.

Phylogenetic relationships of 46 protein-coding genes within Selaginellaceae were basically congruent with the previously published results (Weststrand and Korall 2016a; Zhou et al. 2016) except for the phylogenetic position of *S. sanguinolenta* (fig. 1). *Selaginella sanguinolenta* was placed in the second earliest diverging subg. *Boreoselaginella*, except for the basal group subg. *Selaginella*, and was sister to the rest of the genus in Zhou et al. (2016). In Weststrand and Korall (2016a), *S. sanguinolenta* was found in two different positions (position α and position β), and the phylogenetic position of *S. sanguinolenta* in our newly reconstructed phylogeny was congruent with the position β with 100% support. The results from ancestral states reconstruction (fig. 7) indicated that the DR structure was the ancestral state whereas IR structure was the derived state in sect. *Lepidophylla* and subg. *Heterostachys* (fig. 1) However, the IR structure presented in *S. lepidophylla* seemed to be an exception. The split between Selaginellaceae and its sister family Isoetaceae occurred at 375 Ma (Kenrick and Crane 1997). We inferred that the occurrence time of the DR structure and the recurrence time of the IR structure (IR/DR-coexisting) was at ∼257 and ∼19 Ma, respectively (fig. 1). The split time between *S. lepidophylla* and sect. *Homoeophyllae* (*S. vardei* and *S. indica*) occurred at ∼143 Ma.

## Discussion

### Gene/Intron Losses and Pseudogenes

Compared with *S. uncinata* (Tsuji et al. 2007) and *S. moellendorffii* (Smith 2009), the tRNA loss was even more severe in the newly sequenced *Selaginella* species with only eight different tRNA genes remaining in *S. tamariscina*. However, 22 tRNA genes were annotated in the plastome of *S. sanguinolenta* (table 1 and fig. 3) with the presence of three “vestigial” tRNA genes, which were also found in *S. uncinata* plastomes (seven “vestigial” tRNA genes) (Tsuji et al. 2007). Several hypotheses have been proposed to explain why more than half of tRNA genes has been lost and how to compensate for the absence of tRNA genes from *Selaginella* plastome (Wolfe et al. 1992) (Tsuji et al. 2007). The hypothesis that the lost tRNA are encoded in the nuclear genome and imported to the plastid from the cytosol, which is also known to occur for plant mitochondria, is most likely to be the explanation for the presence of “vestigial” tRNA genes found in *S. sanguinolenta* and *S. uncinata*. Furthermore, no tRNA genes were annotated in mitochondrial genome of *S. moellendorffii* (Hecht et al. 2011). Therefore, the absent tRNA genes in plastomes of *Selaginella* are most likely lost and imported from nucleus.

Except for the severe tRNA genes loss mentioned earlier, numerous protein-coding genes were also absent in *Selaginella* plastomes, especially in the species of dry habitat (fig. 2). Six species had *ndh* gene loss to a different extent with all *ndh* genes lost in *S. vardei*, *S. indica*, *S. lepidophylla*, and *S. lyallii*, 10 lost and 1 (*ndhC*) intact in *S. tamariscina*, 4 lost, 4 pseudogenized, and 3 intact in *S. sanguinolenta*, and 11 *ndh* genes intact in other 7 *Selaginella* species. The gene loss of *ndh* genes has been reported in land plant plastomes for many times, such as, *Najas flexilis* (Peredo et al. 2013), *Epifagus*, (Haberhausen and Zetsche 1994), *Cuscuta* (Funk et al. 2007; Logacheva et al. 2011), *Neottia* (Haberle et al. 2008; Feng et al. 2016), Gnetales (Raubeson and Jansen 2005), Pinaceae (Wakasugi et al. 1994), and several *Erodium* species (Blazier et al. 2011). The n*dh* genes, encoding plastid NAD(P)H–dehydrogenase complex, are involved in cyclic electron flow (CEF) chain. Two independent pathways of CEF, *PGR*5-dependent, and *NDH*-dependent pathways, have been characterized across land plants, with the former being the main contributor in CEF (Ruhlman et al. 2015). Therefore, the *ndh* genes that were lost in different lineages of land plants were not transferred into nucleus, but most possibly replaced by the nuclear-encoded *PGR*5-dependent pathway (Ruhlman et al. 2015). The shared character in *S. vardei*, *S. indica*, *S. lepidophylla*, *S. lyallii*, and *S. tamariscina* is the extremely dry habitat and thick wax-like components on the leaf surface, likely to reflect the strong sunlight. In this case, we presume that the loss of *ndh* genes in these five species could be related to the adaptation to the dry and light-intensive habitat. The plastome of *S. sanguinolenta* could be in the intermediate phase growing in the moderate dry habitat, and the loss and pseudogenization of *ndh* genes could be a relatively recent event. In *S. bisulcata*, all *ndh* genes become putative pseudogenes because of the premature stop codons caused by frame shift mutation, whereas all *ndh* genes are functional in the plastome of its sister species *S. pennata* (fig. 2 and supplementary fig. S3, Supplementary Material online). The extensive RNA editing sites in plastomes of *Selaginella* were previously reported, but only limited to C-to-U editing, which is not able to restore the premature stop codons (Oldenkott et al. 2014). Furthermore, evidence from transcriptome data is necessary to elucidate whether the putative pseudogenes in *S. bisulcata* are truly deprived of function or restored into functional genes at the transcriptome level.

Another noteworthy event is the intron loss in Selaginellaceae (fig. 2). The intron loss in plastomes is common in land plants (Jansen and Ruhlman 2012). Most angiosperms and ferns have two introns in *clpP* gene, whereas only one intron remained in *clpP* gene of Equisetaceae in ferns (Karol et al. 2010). In *Selaginella*, however, two introns remained in *clpP* of *S. sanguinolenta* and *S. doederleinii* as in other lycophytes (Isoetaceae and Lycopodiaceae), only one intron, similar to the plastomes of Equisetaceae, remained in *clpP* gene of other reported *Selaginella* species, and no intron existed in *clpP* of *S. tamariscina*, *S. remotifolia*, *S. kraussiana*, *S. lyallii*, *S. lepidophylla*, *S. indica*, and *S. vardei*, similar to the plastomes of *Pinus* species (Wakasugi et al. 1994), two *Silene* species (Erixon and Oxelman 2008), and grasses (Downie and Palmer 1992; Barkan 2004). The intron of *atpF* gene was lost in *S. vardei* and *S. indica*, while the intron of *rpoC*1 was absent in *S. lyallii*. Besides, the intron 2 of *ycf*3 gene was lost in *S. vardei*, *S. indica*, *S. lepidophylla*, *S. lyallii*, *S. kraussiana*, and *S. remotifolia*. Observation of the intron loss in *atpF* was first uncovered in the plastome of cassava (Daniell et al. 2008) and the loss of *rpoC*1 intron was found to occur multiple times in angiosperms (Downie et al. 1996). The loss of intron 2 of *ycf*3 genes represents the first documented case within the plastomes of land plants. In the case of intron loss, one mechanism has been proposed that involves recombination between a processed intron-less cDNA and the original intron-containing copy (Daniell et al. 2008). Under this situation, an apparent decrease of RNA editing sites in the neighboring regions of lost intron should be observed in genes losing intron. The multiple sequence alignments between intron-lost genes (*atpF*, *clpP*, *rpoC1*, and *ycf3*) and their homologs with intron from closely related species displayed apparent C to T change at the flanking regions of lost introns (supplementary fig. S4, Supplementary Material online). Therefore, we propose that the intron loss of *clpP*, *atpF*, *rpoC*1, and *ycf*3 in Selaginellaceae can likely be explained by this mechanism.

### Evolutionary Trajectory of DR/IR in Selaginellaceae

In addition to the DR structure in *S. indica*, *S. vardei* (Zhang et al. 2018), *S. tamariscina* (Xu et al. 2018), and *S. kraussiana* (Mower et al. 2018), we also found the DR structure in the plastomes of our newly sequenced seven species (*S. lyallii*, *S. remotifolia*, *S. sanguinolenta*, *S. tamariscina*, *S. doederleinii*, *S. moellendorffii*, *S. bisulcata*, and *S. pennata*), which showed that the DR structure in *Selaginella* plastomes is a remarkable character. However, the typical IR structure, in almost all land plants, still remains in the plastomes of *S. lepidophylla*, *S. uncinata*, and *S. hainanensis*. Furthermore, PCR confirmation of the plastome structure in 18 representative species from four subgenera sensu Zhou and Zhang (2015) (supplementary table S2, Supplementary Material online) showed that three subgenera in Selaginellaceae possess plastomes with DR structure, whereas in subg. *Heterostachys* the plastomes evolved into the typical IR structure again (fig. 1 and supplementary fig. S1, Supplementary Material online). Although the IR structure is ubiquitous in plastomes among land plants, the result of ancestral states reconstruction indicated that the DR structure is ancestral status and remained in plastomes of most lineages within Selaginellaceae (fig. 1). Given that plastomes from the other two families, Lycopodiaceae and Isoetaceae, of the lycophytes possess the typical IR structure (Wolf et al. 2005; Karol et al. 2010; Guo et al. 2016; Zhang et al. 2018), the DR structure is further confirmed to have occurred in the Selaginellaceae plastomes after the separation from Isoetaceae (Zhang et al. 2018).

The occurrence of the direction change from IR in Isoetaceae to DR in Selaginellaceae was attributed to an inversion of ∼50-kb fragment *trnF*-*trnN* spanning the complete IR_B_ region (Zhang et al. 2018). *Selaginella lepidophylla* is sister group of *S. vardei* clade, which possess plastomes with DR structure (fig. 1). Exceptionally, plastome of *S. lepidophylla* has IR structure, which is also different from the IR-possessing plastome organization of *S. uncinata* and *S. hainanensis* (Mower et al. 2018). The DR structure is considered to be the ancestral state in *Selaginella* plastomes (fig. 7); therefore, plastome of *S. lepidophylla* should also have experienced the shared ∼50-kb inversion, which is also supported by Mower et al. (2018). The ∼68-kb inversion in plastome of *S. lepidophylla*, compared with *S. vardei*, also spanned one copy of repeat region and hence, switched into IR structure. Both DR and IR structures exist in subg. *Heterostachys*, with the plastomes of *S. bisulcata* and *S. pennata* assembled into DR structure and the plastomes of *S. uncinata* and *S. hainanensis* assembled into IR structure. Both types of plastomes showed divergence from the ancestral DR structure, indicating the plastome structure diverged along with the two clades split. A 20-kb inversion (*trnC*-*psbI*) is shared by the plastomes of the four species, showing this inversion occurred before the divergence. After that, the plastomes of two clades both experienced two independent inversion events, among which an ∼65-kb inversion in *S. uncinata* and *S. hainanensis* spanning one copy of repeat region recovered the IR structure (fig. 1).

### Dynamic Structures of the Plastomes with DR/IR Structure of Selaginellaceae

Recombination-dependent process between homologous repeats is responsible for the highly dynamic structure of plant organelle genomes (plastomes and mitogenomes) (Maréchal and Brisson 2010; Ruhlman et al. 2017). Large repeats (>1 kb) are able to mediate highly frequent, reciprocal recombination intra- or intermolecularly and generally results in approximately equimolar amounts of the parental and recombinant forms (Fauron et al. 1995; Arrieta-Montiel and Mackenzie 2011). In genomes where both IRs and DRs are present, recombination activities in these different orientations will lead to drastically different genome organizations, containing various isomeric forms of the master chromosome and subgenomic molecules(Fauron et al. 1995).

The plastomes of *S. uncinata* and *S. hainanensis* were assembled into an IR structure, which we consider as master chromosome. However, another pair of ∼2.7-kb IRs spanning *psb*K-*trn*Q was identified in the LSC and SSC region, respectively (supplementary fig. S5, Supplementary Material online). Therefore, recombination between IR generates an isomer with altered orientation of one single-copy region, which, in turn, changes the IRs of *psb*K-*trn*Q into direct (isomer I). Recombination between the copies of ∼2.7-kb IR *psb*K-*trn*Q could change the orientation of IR into direct (DR) and generates isomer II. Both isomer I and isomer II could give rise to two subgenomic molecules through the recombination between DRs, respectively (fig. 5*a*).

The plastomes of *S. bisulcata* and *S. pennata* were assembled into DR structure, which was also considered as master chromosome. However, a pair of ∼1.8-kb IRs spanning *psb*K-*trn*Q was identified in the LSC and SSC region, respectively (supplementary fig. S5, Supplementary Material online). Following the recombination activities in *S. uncinata* and *S. hainanensis*, recombination between DR gives rise to two subgenomic molecules, and recombination between the copies of ∼1.8-kb IR *psb*K-*trn*Q change the orientation of DR into inverted (IR), generating isomer I. Recombination between newly created IR in isomer I generates isomer II with altered orientation of one single-copy region, which similarly changes the IRs of *psb*K-*trn*Q into direct (isomer II). Finally, recombination between the copies of DR *psb*K-*trn*Q in isomer II also gives rise to two different subgenomic molecules (fig. 5*b*). Thus, these frequent, reciprocal recombination activities created a dynamic complex heterogeneous population of plastomes (IR/DR-coexisting) in *S. uncinata*, *S. hainanensis*, *S. bisulcata*, and *S. pennata*.

However, as reported in *S. vardei* (Zhang et al. 2018), the plastomes with DR structure could only promote a master chromosome and two sets of subgenomic chromosomes at approximately equal stoichiometries by the recombination between two copies of DR within one plastome or between different molecules (fig. 5*c*). Either the master chromosome or subgenomic chromosomes could form head to tail concatemers of both circular and linear molecules together with branched structures through recombination between DR regions. The existence of subgenomes in species of DR-possessing plastomes have been confirmed using the PacBio reads in *S. tamariscina* (Xu et al. 2018), whereas the existence of the complex heterogeneous population of multipartite plastomes in *S. uncinata*, *S. hainanensis*, *S. bisulcata*, and *S. pennata* still need to be confirmed by long reads from PacBio or Nanopore sequencing. Considering the fact that most land plants possess plastomes with IR structure and only the early diverged lycophyte group Selaginellaceae share DR structure, plastomes with IR structure presumably have more advantageous characters for plants survival and adaptation. The coexistence of the dynamic heterogeneous plastome structures in the derived lineage is possibly in the intermediate stage, which also reached an equilibrium in plastome organization. However, the biological significance behind the diverse plastome structures, especially for adaptation to environments, and the role of nuclear-encoded, plastid-targeted genes, which control the recombination behaviors, are worth further exploration.

### Correlation between Plastome Rearrangements and Repeats in Selaginellaceae

Two main forms of rearrangements, inversions and DR/IR region expansion/contraction, constitute the main rearrangement events in plastomes of Selaginellaceae. Based on the published phylogeny of Selaginellaceae (Weststrand and Korall 2016a; Zhou et al. 2016), plastome organizations of basal lineages (e.g., *S. vardei*, *S. indica*, *S. lyallii*, *S. kraussiana*, *S. remotifolia*, and *S. sanguinolenta*) of Selaginellaceae showed relatively conserved gene order, whereas rearrangements mainly existed in the more evolved lineages (e.g., *S. tamariscina*, *S. doederleinii*, *S. bisulcata*, and *S. uncinata*) (fig. 1 and supplementary fig. S1, Supplementary Material online).

The extensive rearrangement events in plastomes of Geraniaceae have shown to be correlated with high incidence of dispersed repetitive DNA (Weng et al. 2014). The correlation between number of repeats and the rearrangement distances was also detected in Selaginellaceae plastomes (BP, *r* = 0.616, *P* = 0.019; IVs, *r* = 0.701, *P* = 0.005, supplementary fig. S6*a* and *b*, Supplementary Material online), with high frequency of repeats in IR/DR-coexisting plastomes (fig. 4). The results are possibly influenced by phylogenetic signal, since closely related species tend to have similar value for rearrangement distances. The results using PICs method did not show correlation between rearrangement distances and number of repeats (BP, *r*=−0.263, *P* = 0.186; IVs, *r*=−0.325, *P* = 0.137, supplementary fig. S6*c* and *d*, Supplementary Material online). The high *P* value suggested that the analysis possibly has no statistical significance and more data should be added in the future to accurately estimate the correlation. Therefore, the correlation between rearrangement distances and number of repeats receive no solid support in this study, and we infer that the number of repeats in Selaginellaceae are presumably more associated with the structure change from DR to IR/DR coexistence. The coexistence of fewer repeats and DR structure in *Selaginella* plastomes, as reported in *S. vardei* and *S. indica* (Zhang et al. 2018) might have conferred an advantage to maintain the plastome stability and have been selected. On the other hand, the occurrence of repeats, especially the large one (*psb*K-*trn*Q), in the IR/DR-coexisting plastomes are responsible for the hypothesized dynamic plastome complexity, which has reached an equilibrium state in order to maintain stability.

### Correlation between Plastome Rearrangements and Nucleotide Substitution Rate in Selaginellaceae

The nearly similar value of d*S* between genes of DR-possessing and IR-possessing plastomes (fig. 6*a*) showed that the efficiency of recombination activities is basically equivalent in these two types of plastomes. The significantly low d*S* value of genes of IR/DR-coexisting plastomes (fig. 6*a*) indicated that more efficient recombination activities, functioning as gene conversion mechanism and occurring within the single-copy regions, consequently decrease the d*S* value in IR/DR-coexisting plastomes (Ruhlman and Jansen 2014; Ruhlman et al. 2017). However, the genes from IR/DR-coexisting plastomes exhibited accelerated d*N* and d*N*/d*S* compared with the genes of DR-possessing and IR-possessing plastomes (fig. 6*a*) suggesting that genes in species with IR/DR-coexisting plastomes is presumably subject to slightly lower selective pressures, which may require fixation of functionally important mutations (Bousquet et al. 1992). For species with DR-possessing plastomes, the difference of d*S* between genes inside IV and outside IV was not significant (*P* = 0.070) (fig. 6*b*), showing the ∼50-kb inversion causing DR structure did not have significant influence on synonymous substitution rate. The significantly low d*N* value and d*N*/d*S* of genes outside IV (fig. 6*b*) is possibly correlated with the genes themselves, which usually encode the main subunit of photosynthesis-necessary proteins and under strong selection pressure (supplementary table S6, Supplementary Material online).

## Supplementary Material

Supplementary data are available at *Genome Biology and Evolution* online.

## Supplementary Material

Supplementary Data
