# Supplementary material for: The Unique Evolutionary Trajectory and Dynamic Conformations of DR and IR/DR-Coexisting Plastomes of the Early Vascular Plant Selaginellaceae (Lycophyte)
Source: Genome Biol Evol. 2019 Apr 1;11(4):1258–74. doi: 10.1093/gbe/evz073 (PMC6486807; doi:10.1093/gbe/evz073)
Supplement: Supplementary Data [file evz073_supp.docx]

**Supplementary Material**

**Figure S1. Detailed plastid genome structures of *Selaginella* species. HS**, *H. serrata*; **IF**, *Isoetes flaccida*; **SLe,** *S. lepidophylla*; **SV**, *Selaginella vardei*; **SI**, *S. indica*; **SLy**, *S. lyallii*; **SK,** *S. kraussiana*; **SR**, *S. remotifolia*; **SS**, *S. sanguinolenta*; **ST**, *S. tamariscina*; **SD**, *S. doederleinii*; **SM**, *S. moellendorffii*; **SP**, *S. pennata*; **SB**, *S. bisulcata*; **SU**, *S. uncinata*; **SH**, *S. hainanensis*. Genes are colored by function. Black star marks the occurrence of DR structure, grey star marks the occurrence of IR/DR-coexisting structure, and the hollow grey star marks the IR structure in *S. lepidophylla* in Selaginellaceae. Orange arrows in DR/IR region of plastomes show the contraction and expansion.

**Figure S2 PCR gels of the representative species in subgenera.** **a**, species from subg. *Heterstachys*. **8203**, *S. helferi*; **7833**, *S. picta*; **2415**, *S. mairei*; **7187**, *S. delicatula*; **6548**, *S. willdenowii*. **1**, *ycf*2-*ndh*J; **2**, *rpl*2-*rpl*23; **3**, *ycf*3-*rps*4; **4**, *trn*D-*pet*N; **5**, *ndh*F-*rps*4; **6**, *rpl*2-*rps*7. **b**, species from subg. *Stachygynandrum*. **7867**, *S. commutata*; **7899**, *S. guihaia*; **7895**, *S. rolandiprincipis*; **8176**, *S. scabrifolia*; **7863**, *S. biformis*; **7643**, *S. davidii*; **8201**, *S. erythropus*; **2016015**, *S. gebauriana*; **6050**, *S. involvens*. **1**, *ycf*3-*rps*4; **2**, *rrn*16-*rpl*23; **3**, *trn*F-*chl*L; **4**, *ndh*F-*rps*4; **5**, *rrn*16-*rps*7. **c**, species from subg. *Pulviniela*. **519**, *S.* *pulvinata*; **7644**, *S. stauntoniana*. **1**, *ycf*3-*ycf*3; **2**, *rrn*16-*rpl*23; **3**, *ndh*C-*chl*L; **4**, *ccs*A*-ycf*3; **5**, *rrn*16-*rps*7. **d**, species from subg. *Boreoselaginella*. **179**, *S. nummularifolia*; **7833**, *S. rossii*. **1**, *rps*4-*rrn*5; **2**, *ndh*B-*rpl*2; **3**, *trn*F-*chl*L; **4**, *ndh*F-*rrn*5; **5**, *trn*L-*trn*C.

**Figure S3 Comparisons of *ndh* genes between *S. bisulcata* and *S. pennata*.**

**Figure S4 Comparisons of intron-loss genes among closely related species.**

**Figure S5. Distribution of repeats in plastomes of newly sequenced *Selaginella* species.**

**Figure S6. Correlation between rearrangement distance and repeats in Selaginellaceae. a**, correlation analysis between number of repeats and BP; **b**, correlation analysis between number of repeats and IVs; **c,** correlation analysis between number of repeats and BP using PICs method; **d**, correlation analysis between number of repeats and IVs using PICs method.

**Supplementary tables:**

Table S1. Technical details of the Illumina datasets and genome assemblies.

Table S2 PCR confirmation for plastomes structure of representative species of four subgenera.

Table S3. Primers newly designed for PCR amplifications of the sampled representative species of the five main lineages in Selaginellaceae.

Table S4. The permutation of number coded Locally Collinear Block (LCB) for each plastome. Negative number indicates an inversion of the given LCB.

Table S5. Locally Collinear Blocks identified by Mauve alignment of 12 plastomes of lycophytes.

Table S6. Genes inside and outside ca. 50 kb inversion of plastomes with DR structure.
